# Total knee arthroplasty improves gait adaptability in osteoarthritis patients; a pilot study

**DOI:** 10.1016/j.jor.2022.08.003

**Published:** 2022-09-05

**Authors:** M.J. Booij, B.J. van Royen, P.A. Nolte, J.W.R. Twisk, J. Harlaar, J.C. van den Noort

**Affiliations:** a Amsterdam UMC, Vrije Universiteit Amsterdam, Department of Rehabilitation Medicine, Amsterdam Movement Sciences, de Boelelaan, 1117, Amsterdam, the Netherlands; b Amsterdam UMC, University of Amsterdam and Vrije Universiteit Amsterdam, Department of Orthopaedic Surgery, Amsterdam Movement Sciences, Meibergdreef 9, Amsterdam, the Netherlands; c Spaarne Gasthuis, Department of Orthopaedic Surgery, Spaarnepoort 1, Hoofddorp, the Netherlands; d VU University Medical Centre, Department of Epidemiology and Biostatistics, Amsterdam, the Netherlands; e Delft University of Technology, Department of Biomechanical Engineering, Mekelweg 2, Delft, the Netherlands; f Erasmus Medical Center, Department of Orthopedics and Sports Medicine, Dr. Molewaterplein 40, Rotterdam, the Netherlands; g Amsterdam UMC, University of Amsterdam, Department of Radiology and Nuclear Medicine, Medical Imaging Quantification Center, Amsterdam Movement Sciences, Meibergdreef 9, Amsterdam, the Netherlands

**Keywords:** Walking adaptability, Stepping stones, Total knee replacement, Aging, Motor control

## Abstract

**Background:**

Gait adaptability is of utmost importance for keeping balance during gait in patients with knee osteoarthritis, also after total knee arthroplasty (TKA). The aims of this explorative study are: (1) assess the effect of age, knee osteoarthritis and TKA on gait adaptability; (2) assess changes in gait adaptability pre-to post-TKA and (3) their relation to functional outcomes.

**Methods:**

Gait adaptability was measured using a Target Stepping Test (TST) in knee osteoarthritis patients before (preTKA) and 12 months after TKA (postTKA) and compared to asymptomatic old (AsOld) and young adults (AsYng). TST imposed an asymmetrical gait pattern with projected stepping targets at high walking speed. Gait adaptability was determined through stepping accuracy on the targets. The Oxford Knee Score (OKS) and Timed-Up-and-Go test (TUG) measured patients’ physical function.

**Results:**

12 preTKA, 8 postTKA, 18 AsYng, 21 AsOld were tested. Age showed no effect on TST-stepping accuracy. PreTKA showed worse TST-stepping accuracy compared to AsYng and AsOld (7.7; 6.2 cm difference). PostTKA showed an improvement of 52% in TST-stepping accuracy compared to preTKA (3.2 cm).

Higher stepping accuracy preTKA predicted higher stepping accuracy post-TKA. In addition, low preTKA stepping accuracy predicted more improvement postTKA. Pre-to post-TKA improvement of stepping accuracy was related to improvement on the TUG (Beta = 0.17, p = 0.024), but not to OKS.

**Conclusions:**

Gait adaptability is improved following TKA in knee osteoarthritis patients and no longer significantly worse than asymptomatic adults. The relation of gait adaptability to function is shown by its relation to the TUG and shows to have predictive value pre-to post-TKA.

## Highlights

• Gait adaptability can be measured through stepping accuracy on stepping targets.
• Gait adaptability is related to physical function on the Timed-Up-and-Go Test.
• Gait adaptability deteriorates with the development of knee osteoarthritis.
• After total knee replacement gait adaptability is greatly improved.
• Gait adaptability may predict functional improvement after total knee arthroplasty.

## 1 Introduction

Elderly, especially people suffering from osteoarthritis, are at high risk of falling. More than half of knee osteoarthritis patients experience a fall each year, which is twice as much as the average older population.^1^ While some risk factors have been identified, no effective strategy to reduce fall risk is shown so far.^2^ Therefore, causal factors need to be identified to be able to reduce occurrence of these events.

Risk factors for falls are loss of self-efficacy, limited proprioception, muscle weakness, instability and inactivity.^1^^,^^3^^,^^4^ These factors are also known to compromise gait adaptability in older adults^5^^,^^6^ and are significantly worsened due to knee osteoarthritis,^3^ further increasing the risk of falls.^7^ Up to 56% of older adults receive treatment for a fracture after a fall and 40% of older adults report a decline in function after a fall.^8^^,^^9^

Motor control is defined as the ability to regulate mechanisms essential to movement.^10^ Gait adaptability is a key expression of motor control, and is defined as the ability to adapt the walking pattern to environmental circumstances.^11^ Especially timely and precisely placement of the foot, accounting for the position of the body center of mass and its dynamics, is critical to maintain balance during gait. Most falls during walking are caused by insufficient gait adaptability.^12^ Moreover, an increased risk of falling in elderly is related to reduced gait adaptability as measured by stepping accuracy.^13^, ^14^, ^15^ As a consequence, sufficient gait adaptability to cope with environmental challenges is of utmost importance to lower fall risk. Until now the Timed-Up-and-Go test and the Oxford Knee Score are the only clinical measures of physical function that have been related to fall risk^16^ or gait biomechanics.^15^^,^^24^ A clinical measurement of gait adaptability may be an informative addition to understand elevated fall risk or pathological gait biomechanics.

To assess gait adaptability a widely applied method using stepping targets could be used. Such a method resembles daily life situations in which goal-directed stepping strategies are needed and allows for calculation of a sensitive stepping accuracy score. Moreover, target stepping assessments are feasible tests that can be applied using commercially available systems.^17^^,^^18^

Since knee osteoarthritis increases fall risk,^7^ which is likely to be mediated by decreased gait adaptability, along with other musculoskeletal impairments that worsen mobility and functioning. Treatment of knee osteoarthritis with total knee arthroplasty (TKA) improves functioning and lowers fall risk.^2^^,^^4^ More specifically, TKA alleviates pain and improves joint range of motion, which have been related to decrease of fall risk.^19^ Pain and range of motion are factors that regulate adaptation of body movement, while control over such movement is necessary to employ these gait adaptations. This suggests that TKA also improves gait adaptability as a working mechanism in reducing fall risk, but this has not been shown in previous literature. Knowledge of this additional benefit of TKA for the patient, can inform clinical decision-making. It suggests that knee osteoarthritis patients with elevated fall incidence could experience additional benefit from surgery. Furthermore, gait adaptability might be trained and used as a targeted therapeutic intervention to reduce fall risk.

Hence, this pilot study aims to: (1) explore the effect of age, knee osteoarthritis and TKA on gait adaptability; (2) assess how gait adaptability is affected after TKA; and to(3) investigate the relationship between gait adaptability and clinical function scores. We hypothesize that: (1) aging will reduce gait adaptability, with further reduction in presence of severe knee osteoarthritis; (2) patients will improve their gait adaptability post-TKA; and (3) we hypothesize greater effect sizes in case of worse functioning pre-TKA.

## 2 Materials and methods

### 2.1 Participants

Twelve symptomatic older patients with severe knee osteoarthritis indicated for TKA (preTKA) were included, aged 50–75 years. 18 asymptomatic young adults (AsYng) under 25 years old were also included, as well as 21 asymptomatic older adults (AsOld) that were age-matched to the preTKA group. The preTKA group was planned for primary total knee arthroplasty (TKA) at inclusion and underwent a follow-up measurement one year post-TKA (postTKA). All participants could walk without aids, did not have comorbidities that could affect the gait pattern, such as diagnosed osteoarthritis, neurological disorders or a prosthesis in any lower limb joint (other than the unilateral knee osteoarthritis or replacement for patients). Ethics approval was granted from the local Human Research Ethics Committee of the Amsterdam UMC, location VUmc on 22 February 2019 (NL67077.029.18), and written informed consent was provided according to the Committees’ guidelines.

### 2.2 Data collection

Data were collected on the GRAIL (Gait Real-time Analysis Interactive Lab, Motek ForceLink BV, Netherlands) at the Amsterdam UMC, location VUmc. Participants walked on an instrumented treadmill in a virtual environment (a forest trail). During the walking trials, 3D motion was captured via InfraRed optical motion capture with wireless, light-reflecting markers (Vicon version 2.5, Oxford, UK). For this study, 26 markers were placed on the subject for reconstruction of the position and orientation of the lower limbs, pelvis and trunk in space, according to the CAST model,^20^ recorded at a 100Hz sample frequency.

Foot length was defined as the length of the line between the markers at the toe and the calcaneus. Midfoot location was determined as halfway the foot length.

Comfortable walking speed (CWS) was determined during 3 min of self-paced walking, during which the treadmill adapted its speed to the location of the participant on the treadmill. This followed by a minimum of 5 min of habituation to treadmill walking in which during the last half minute step length and step width were determined. The following 30 s participants practiced walking on stepping targets projected on the treadmill. Stepping targets were projected, while participant still walked at CWS. Targets matched length and width of the feet and were projected at matching step length and step width as recorded during the previous comfortable walking trial. Participants could see up to six targets ahead. Participants were instructed to step as precisely as possible on the stepping targets. Real-time stepping accuracy was determined by calculating the stepping error, which is the distance between midpoint target and midfoot (midpoint of the foot was calculated as halfway between the toe and calcaneus marker on the anteroposterior axis and halfway between the MTP1 and MTP5 marker in mediolateral axis). If the stepping error was less than 2 cm in each direction, the projected stepping target turned green and a piano tone was heard.

**Fig. 1:**
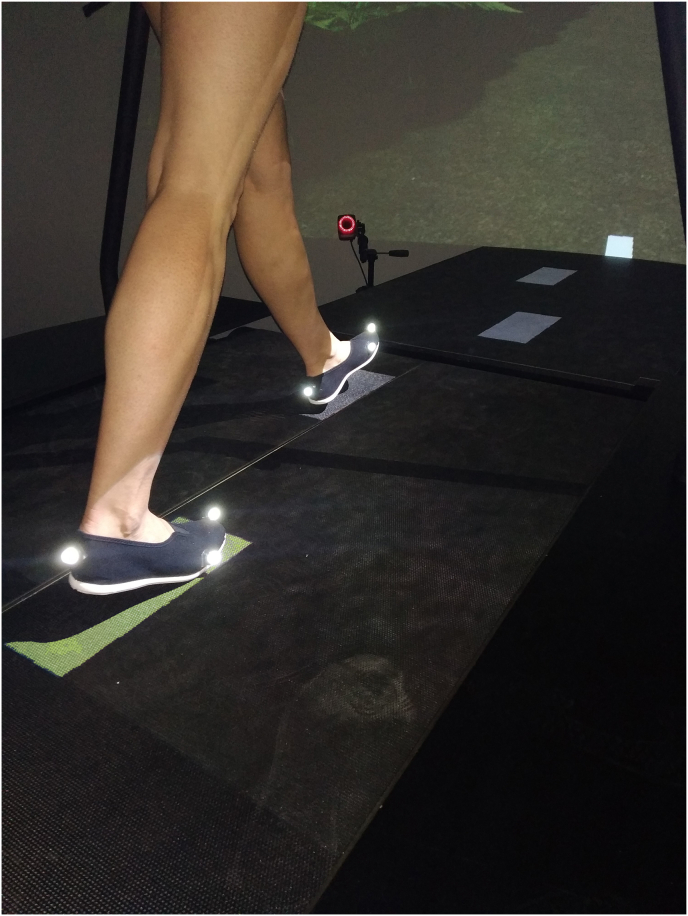
Target Stepping Test Targets were presented up to 6 targets ahead. In this example the left foot targets imposed 20% shorter step length, and right foot targets imposed 20% longer step length.

Finally, the Target Stepping Test (TST) started (Fig. 1). This test imposed a constant asymmetric gait pattern for 3 min, with on one side 20% shorter step length (for AsYng and AsOld at random side, for patients their symptomatic and later operated side) and the other side a 20% longer than baseline step length. The test was performed at 130% of comfortable walking speed to impose sufficient challenge.

For the patients, additional clinical function measures were taken. Patients filled out the Oxford Knee Score (OKS) to determine self-perceived function^21^ and performed the Timed-Up-and-Go test (TUG) as a quantitative clinical function measure.^22^

### 2.3 Data processing

Stance phase was determined between initial contact and toe-off using the marker data following the method of Zeni et al.^23^ based on foot velocity. Then, average stepping accuracy per step was determined over the midstance period (20–30% of the stance phase) in the anteroposterior direction. Finally, average stepping accuracy was calculated over the last 80 steps of the test.

The concept of gait adaptability is expressed in stepping accuracy which is measured by the magnitude of the stepping error (the difference between midfoot and midpoint target in cm), which definitions are inversely related: a worse stepping accuracy is shown by a greater stepping error.

### 2.4 Statistical analysis

Normality of all outcome parameters was confirmed using visual inspection and the Shapiro-Wilk test.

First, the effects of age, osteoarthritis and total knee arthroplasty on stepping accuracy were determined. To do so, group differences (AsYng, AsOld, preTKA, postTKA) were determined using a linear mixed model analysis, taking into account the dependency of the observations within the patient preTKA and postTKA.

Second, linear regression analyses were used to analyze the relationship between stepping accuracy pre-TKA and stepping accuracy post-TKA and between stepping accuracy pre-TKA and the change in stepping accuracy between pre- and post-TKA.

Third, linear regression analyses were used to analyze the relationship between stepping accuracy pre-TKA and the two clinical function scores (OKS and TUG) post-TKA and between stepping accuracy pre-TKA and the change in pre-to post-TKA function scores. Moreover, a linear regression analysis was performed between the change in pre-to post-TKA stepping accuracy and the change in pre-to post-TKA function scores.

All statistical analyses were corrected for walking speed.

All analyses were carried out in MATLAB R2018b (MathWorks, Natick, MA) and STATA 17.0 (Statacorp, College Station, TX). Significance was set at alpha 0.05.

## 3 Results

Twelve older patients with severe unilateral knee osteoarthritis (preTKA), eighteen asymptomatic young adults (AsYng) and 21 asymptomatic older adults (AsOld) were included (Table 1). Eight patients from the preTKA group were available for follow-up measurements after their total knee arthroplasty (postTKA). One patient withdrew from a total knee arthroplasty; 3 follow-up measurements were cancelled due to restrictions caused by the Covid-19 pandemic.

**Table 1:** **Demographics** Mean ± SD.

Group	N	Gender (n)	Age (years)	BMI (kg/m^2^)	130% CWS (m/s)
AsYng	18	9 male	23.3 ± 1.8	22.7 ± 3.0	1.68 ± 0.21
AsOld	21	13 male	66.4 ± 5.5	25.6 ± 3.2	1.57 ± 0.21
preTKA	12	9 male	67.3 ± 5.4	28.6 ± 3.5	1.38 ± 0.19
postTKA	8	6 male	68.8 ± 3.8	30.2 ± 3.8	1.38 ± 0.16

No significant difference in age was present between AsOld and preTKA or postTKA (p > 0.05). All groups had significant differences in BMI (p < 0.05), except for preTKA and postTKA (p > 0.05). Each of the asymptomatic groups (AsYng and AsOld) walked significantly faster than the patients (p < 0.002).

### 3.1 Effect of age, knee osteoarthritis and total knee arthroplasty on stepping accuracy

**Fig. 2:**
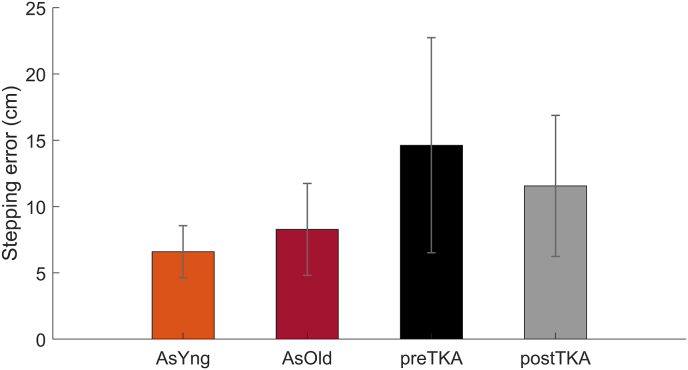
**Stepping accuracy per group** expressed in stepping error. Higher error means a worse stepping accuracy.

AsYng did not show a significantly better stepping accuracy than AsOld (mean difference 1.6 cm, p = 0.275), but did show better stepping accuracy than preTKA (7.7 cm, p < 0.001) and postTKA (4.6 cm, p = 0.008). AsOld showed better stepping accuracy than preTKA (6.2 cm, p < 0.001), but not better than postTKA (3.0 cm, p = 0.053). Compared to AsOld, preTKA showed 77% worse stepping accuracy, while at postTKA the stepping accuracy was only 39% worse than AsOld. Stepping accuracy did improve significantly from preTKA to postTKA (3.2 cm, p = 0.016) (Fig. 2 and Table 2), which means a recovery of 52% due to the TKA.

**Table 2:** Group Comparisons of average stepping accuracy corrected for walking speed. Stepping accuracy is expressed as stepping error in centimeters.

Mean group error			Mean difference (Speed corrected)	95% confidence interval		
	Group comparisons			lower	upper	p-value
6.6	AsYng	AsOld	1.6	−1.2	4.4	0.275
		preTKA	7.7	4.2	11.3	**<0.001**
		postTKA	4.6	1.2	7.9	**0.008**
8.3	AsOld	preTKA	6.2	3.0	9.4	**<0.001**
		postTKA	3.0	−0.0	6.0	0.053
14.7	preTKA	postTKA	3.2	0.6	5.8	**0.016**
11.4	postTKA					

Significant group differences are indicated in bold p-values (p < 0.05).

### 3.2 Prediction stepping accuracy before and after TKA

**Fig. 3:**
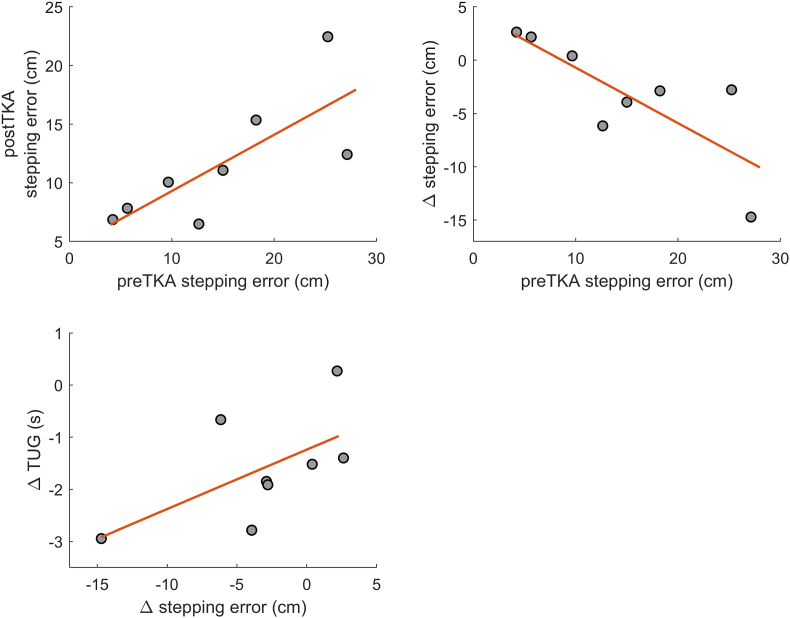
**Significant relationships between stepping accuracy (stepping error) pre- and post-TKA and with clinical function scores.** Shown regression lines are not corrected for speed. The analyses were corrected for speed. Δ = difference between post- and pre-TKA score, a negative Δ is an improvement. Stepping accuracy is expressed as the stepping error, which definitions are expressed inversely.

Stepping accuracy pre-TKA linearly predicted the stepping accuracy post-TKA (Beta = 0.48, p = 0.010) (Table 3 and Fig. 3). The higher the stepping accuracy (as measured through a lower stepping error) pre-TKA, the better the stepping accuracy is post-TKA.

**Table 3:** Relation stepping accuracy and clinical function.

Independent	Dependent	Beta Coefficient	95% confidence interval			
			SEM	lower	upper	p-value
preTKA stepping accuracy	postTKA stepping accuracy	0.48	0.12	0.17	0.78	**0.010**
preTKA stepping accuracy	Δ stepping accuracy	−0.52	0.12	−0.83	−0.22	**0.007**
						
preTKA stepping accuracy	postTKA TUG	−0.04	0.05	−0.16	0.07	0.370
preTKA stepping accuracy	Δ TUG	−0.09	0.04	−0.19	0.01	0.081
						
preTKA stepping accuracy	postTKA OKS	−0.37	0.19	−0.81	0.07	0.090
preTKA stepping accuracy	Δ OKS	−0.30	0.44	−1.30	0.71	0.518
						
Δ stepping accuracy	Δ TUG	0.17	0.05	0.03	0.31	**0.024**
Δ stepping accuracy	Δ OKS	0.69	0.68	−1.05	2.43	0.356

Δ = difference between post- and pre-TKA score. Significant regressions are indicated in bold p-values (p < 0.05).

Moreover, stepping accuracy pre-TKA was inversely associated with the change in stepping accuracy pre-to post-TKA (Beta = −0.52, p = 0.007) (Table 3). This indicates that a lower stepping accuracy pre-TKA, predicts a greater improvement post-TKA.

### 3.3 Relation stepping accuracy and clinical function scores

No significant relations were found between the stepping accuracy and scores on the OKS. The change in stepping accuracy pre-to post-TKA was significantly associated with the change in TUG scores pre-to post-TKA (Beta = 0.17, p = 0.024) (Fig. 3, Table 3).

## 4 Discussion

This pilot study showed that gait adaptability is worsened by knee osteoarthritis and is improved after total knee arthroplasty. Age had no significant impact on gait adaptability. Over half of the decline in gait adaptability due to knee osteoarthritis was restored after TKA. Moreover, the level of gait adaptability before TKA predicts gait adaptability after TKA. In addition, difference in gait adaptability is related to the difference in functional performance pre-to post-TKA, expressed by the Timed-Up-and-Go (TUG) test. Gait adaptability increases after TKA, which in turn increases walking stability, possibly reducing the risk of falling. The predictive value of gait adaptability following TKA could help in clinical decision-making concerning surgical treatment in osteoarthritis and will manage patient expectations for the outcome of surgery.

### 4.1 Effect of age, knee osteoarthritis and total knee arthroplasty on gait adaptability

Contrary to our hypothesis older adults did not show a significantly worse average stepping accuracy compared to young adults. Previous studies did show age-related differences in stepping accuracy.^13^^,^^24^ In our study, older adults were on average 66 years old and relatively younger in comparison to previous studies (average 71 (N = 8) and 74 (N = 50) years old). This might explain why no significant effect of age on gait adaptability was found in our study.

In line with our hypothesis, the patients with knee osteoarthritis (preTKA) had significantly worse stepping accuracy than their age-matched older adults (AsOld), while the lower walking speed of the preTKA group could have made accurate stepping easier. Following TKA, the average stepping accuracy improved significantly by 22% compared to before TKA. After TKA the average stepping accuracy was no longer significantly different from older adults. Moreover, TKA recovers the reduction in gait adaptability due to knee osteoarthritis by 52% compared to asymptomatic old adults. Thus, TKA restores more than half of the negative impact of knee osteoarthritis on gait adaptability.

As fall risk is related to gait adaptability,^13^^,^^14^ these two function measures may correlate in our population. Fall risk is known to increase in older adults and to further increase when severe knee osteoarthritis develops, and to reduce again after TKA.^2^ This difference in fall risk between asymptomatic, knee osteoarthritis and after TKA is similar to the difference in gait adaptability over these three groups found in our study. This study demonstrates that in patients with severe knee osteoarthritis gait adaptability is 77% worse than asymptomatic controls. Moreover, annual fall incidence for severe knee osteoarthritis patients is 67% greater than asymptomatic controls.^3^ Further, this study showed that gait adaptability greatly improved after TKA by 52%. This is in alignment with the review of Frattura et al.^2^ showing that pre-operative fall prevalence ranged from 23% to 63%, while post-operative values ranged from 12% to 38%, which is about half of the level before TKA. These numbers are comparable to the reduction of 39% in stepping accuracy after TKA in our study. These findings point to an equal amount of improvement in gait adaptability as reduction in falls after TKA. Therefore, gait adaptability changes between asymptomatic, knee osteoarthritis and TKA patients, follow the same pattern as fall risk.

### 4.2 Prediction stepping accuracy before and after TKA

Gait adaptability showed to have predictive value to the functional outcome of surgery. The level of gait adaptability before TKA was related to the amount of improvement in stepping accuracy pre-to post-TKA, but also to the absolute value of stepping accuracy after TKA. Patients with a high stepping accuracy before TKA also showed high stepping accuracy after TKA. These outcomes suggest that the gait adaptability before TKA dictates the outcome of gait adaptability after TKA. Part of the postTKA group showed worse gait adaptability than any of the control subjects, which may lead to worse walking stability and increased fall-risk. Current standardized training programs for osteoarthritis and TKA do not include gait adaptability training. Future research should investigate the benefit of gait adaptability training to clinical outcomes to determine whether gait adaptability training should become standard practice. Such training should either be part of pre-rehabilitation programs^25^ or as part of rehabilitation after TKA. This additional training should be considered for patients with worse gait adaptability, to achieve a greater improvement of gait adaptability pre-to post-TKA.

### 4.3 Relation stepping accuracy and clinical function scores

Finally, the relation of gait adaptability to clinical function scores was determined. No relationship of stepping accuracy to the Oxford Knee Score (OKS) was found. However, there was a relationship between the changes pre-to post-TKA in stepping accuracy and the change in Timed-Up-and-Go (TUG) score. This finding is in line with finding within patients suffering from Parkinson's disease.^18^ Greater improvement in stepping error pre-to post-TKA is correlated to greater improvement in performance on the TUG. More specifically, if the stepping error reduces 10 cm pre-to post-TKA, the TUG can be performed 1.7 s faster after TKA (a Beta of 0.17). Previous research has found relations between gait biomechanics and the OKS.^15^^,^^24^ Apparently, stepping accuracy measures another concept of physical function than measured by OKS, while function as measured by TUG is more related to gait adaptability. Performance-based and patient-reported measures could measure different constructs of function.^26^ Both the TUG and gait adaptability are related to fall risk.^14^, ^15^, ^16^ The relation of stepping accuracy to TUG shows that this measure of gait adaptability does have a relation to physical function and can be used as an objective construct.

### 4.4 Limitations

The small sample size in this pilot study should be kept in mind when interpreting the results, especially for the patient group. Instead, significant findings in such low numbers, can only be explained by the large effect size. Nevertheless, current outcomes should be viewed as exploratory, and future research should confirm the effect of knee osteoarthritis and TKA on gait adaptability in larger sample sizes. Furthermore, the relationship between the stepping error and TUG difference pre-to post-TKA seems mainly determined by one greatly improving patient. Therefore, future research should aim to include patients with a larger variety in the amount of improvement pre-to post-TKA to confirm our findings.

While this study showed improved gait adaptability after TKA, the effect could have been affected by the Covid-19 pandemic. The recovery period of patients took place during two lockdown periods. Patients reported to have difficulty keeping up with their daily movement targets and physiotherapy meetings were sometimes postponed.

All regression analyses relations with stepping accuracy improved when corrected for walking speed. Future research investigating stepping accuracy could consider measuring the Target Stepping Test at the same speed for all patients.

Finally, including fall incidence data for the participants would allow a more direct comparison between the two concepts of fall risk and gait adaptability.

## 5 Conclusions

TKA improves gait adaptability in patients with severe knee osteoarthritis. Severe knee osteoarthritis patients showed 77% worse gait adaptability compared to asymptomatic adults. From pre-to post-TKA, gait adaptability was improved by 22% and gait adaptability after TKA was no longer significantly worse than for asymptomatic adults. Hence, our study showed that 52% of the reduction in gait adaptability due to the development of severe knee osteoarthritis is restored after TKA. Further, as shown by the relation between TUG and stepping error, this measure of gait adaptability has a relation to physical function as an objective construct. Finally, gait adaptability before TKA has predictive value for the outcome after TKA.

## Funding

This work was supported by the Dutch Arthritis Society (ReumaNederland), grantnumber LLP-23. There was no involvement of the sponsors in this study.

## Author contributions

Conceptualization; MB, JN, JH, BR. Data curation; Formal analysis; Investigation; Project administration; Software; Validation; Visualization; MB. Funding acquisition; BR. Methodology; MB, JT. Supervision; JN, JH, BR. Roles/Writing - original draft; MB, JN. Writing - review & editing; MB, JH, BR, JT, PN, JN.

## Declaration of competing interest

None.
